# Screening for HBV, HCV, HIV and syphilis infections among bacteriologically confirmed tuberculosis prisoners: An urgent action required

**DOI:** 10.1371/journal.pone.0221265

**Published:** 2019-08-22

**Authors:** Marco Antonio Moreira Puga, Larissa Melo Bandeira, Mauricio Antonio Pompilio, Grazielli Rocha de Rezende, Luana Silva Soares, Vivianne de Oliveira Langraf de Castro, Tayana Serpa Ortiz Tanaka, Gabriela Alves Cesar, Sandra Maria do Valle Leone de Oliveira, Sheila Araújo Teles, Renata Terumi Shiguematsu Yassuda, Sabrina Moreira dos Santos Weis-Torres, Sarlete Ferreira Basílio, Julio Croda, Ana Rita Coimbra Motta-Castro

**Affiliations:** 1 Federal University of Mato Grosso do Sul, Campo Grande, MS, Brazil; 2 Oswaldo Cruz Foundation, Mato Grosso do Sul, Campo Grande, MS, Brazil; 3 School of Nursing, Federal University of Goiás, Goiânia, GO, Brazil; 4 Division of Health, State Agency of the Administration of Prisons, Campo Grande, MS, Brazil; Centers for Disease Control and Prevention, UNITED STATES

## Abstract

Viral hepatitis, syphilis, HIV, and tuberculosis infections in prisons have been identified globally as a public health problem. Tuberculosis (TB) and viral hepatitis co-infection may increase the risk of anti-tuberculosis treatment-induced hepatotoxicity, leading to the frequent cause of discontinuation of the first-line anti-tuberculosis drugs. Therefore, the aim of this cross-sectional study was to investigate the epidemiological features of HCV, HBV, syphilis and HIV infections among bacteriologically confirmed tuberculosis prisoners in Campo Grande (MS), Central Brazil. The participants who agreed to participate (n = 279) were interviewed and tested for the presence of active or current HCV, HBV, syphilis and HIV infections. The prevalence of HCV exposure was 4.7% (13/279; 95% CI 2.2–7.1). HCV RNA was detected in 84.6% (11/13) of anti-HCV positive samples. Out of 279 participants, 19 (6.8%; 95% CI 4.4–10.4) were HIV co-infected, 1.4% (4/279, 95% CI 0.5–3.8) had chronic hepatitis B virus (HBsAg positive) and 9.3% (26/279, 95% CI 6.4–13.4) had serological marker of exposure to hepatitis B virus (total anti-HBc positive). The prevalence of lifetime syphilis infection (anti-*T*. *pallidum* positive) was 10% (28/279, 95% CI 7.0–14.2) and active syphilis (VDRL ≥ 1/8 titre) was 5% (14/279, 95% CI 2.9–8.3). The prevalence of TB/HCV co-infection among prisoners with HIV (15.8%) was higher than among HIV-non-infected prisoners (3.8%; *P*<0.05). These results highlight the importance of hepatitis testing among prisoners with bacteriologically confirmed case of TB who can be more effectively and safely treated in order to reduce the side effects of hepatotoxic anti-TB drugs.

## Introduction

Brazil is classified as the third country with the largest prison population in the world [1, 2]. While the other four countries that most imprisoned from 2010 to 2015 presented a reduction in the evolution of the incarceration rate, Brazil exacerbated this number along with its crime statistics. In 2015, Brazil had about 698,618 incarcerated individuals in penitentiary systems (342/100 thousand inhabitants), which are only designed to hold 371,201 [1], classified as the third country with the largest prison population in the world [1, 2]. The state of Mato Grosso do Sul (MS) in Central Brazil has the highest rate of incarceration in the country, with approximately 15,700 inmates [1].

Prisoners are a high-risk group for contracting and transmitting infectious diseases, including tuberculosis (TB), human immunodeficiency virus (HIV), hepatitis B virus (HBV), hepatitis C virus (HCV), and syphilis. This is due in part to confinement related conditions and also to greater socioeconomic disadvantage, substance abuse, and high-risk sexual behaviors within this population before, during, and after incarceration [3].

Tuberculosis (TB) remains one of the main causes of death due to infectious diseases. In 2016, there were 10.4 million new TB cases worldwide [4]. Globally, prisons are directly linked to high prevalence and incidence of TB morbidity and mortality of TB infection compared with other population cohorts [3]. Based on Brazilian TB notification database and incarceration data, it was found that prisons represent a growing proportion of the national TB burden [5, 6]. Although prison infrastructure and conditions vary considerably in different countries, overcrowding and poorly ventilated custodial settings are common, increasing the transmission risks of TB among prisoners [7, 8, 9]. A recent study demonstrated that during 2009–2014, tuberculosis (TB) cases among prisoners rose 28.8% [5].

Although the high-risk of active TB infection in prisoners is well known, the prevalence of HCV, HBV, and syphilis infections among them has not been widely investigated [10]. In addition, the presence of chronic liver disease caused by hepatitis C and B virus is an additive risk factor for the development of anti-TB drug-induced hepatotoxicity (DIH) during anti-TB therapy [11, 12, 13, 14]. Hepatotoxicity is a frequent cause of the discontinuation of three of the first-line anti-tuberculosis drugs [15, 16]. Unfortunately, treatment of chronic HCV and HBV infections, HIV, syphilis, and TB are often not properly administered to Brazilian prisoners because of the numerous difficulties in screening, management and follow-up. Therefore, this study aimed to investigate the epidemiological features of HCV, HBV, syphilis and HIV infections in bacteriologically confirmed tuberculosis prisoners in Campo Grande (MS), Central Brazil.

## Materials and methods

This cross-sectional survey was conducted among male prisoners with bacteriologically confirmed tuberculosis in the capital city of Campo Grande, MS, Central Brazil. From May 2014 to March 2017, male prisoners were recruited from two closed penal institutions: Instituto Penal de Campo Grande (IPCG) and Estabelecimento Penal Jair Ferreira de Carvalho (EPJFC). Based on the latest Census of Prison Units and Aggregate Data, published in 2016, there were approximately 3,400 inmates in these two major penal institutions, which had a capacity to house up to 904 prisoners [17].

All prisoners diagnosed with bacteriologically confirmed TB by the infectious diseases specialists were included in the eligible population. Bacteriologically confirmed case of TB was defined as the presence of at least one positive smear microscopy or solid culture for *Mycobacterium tuberculosis*.

Of the 279 individuals that agreed to participate, 75 individuals were from IPCG (Instituto Penal de Campo Grande) and 204 were from EPJFC (Estabelecimento Penal Jair Ferreira de Carvalho). Participants were informed about the survey, signed a written and informed consent, and then they were interviewed face-to-face to obtain information on sociodemographic characteristics and risk factors using a questionnaire (–S1 and S2 Files). Blood samples were collected from all participants and stored at -70°C. This survey has been approved by the Ethical Committee of the Federal University of Mato Grosso do Sul, Campo Grande, MS, Brazil (CAAE: 32447814.9.0000.0021). Participation of eligible individuals was voluntary and no compensation was provided. The available medical treatment offered to the subjects was the same regardless of whether they participated in the study or not.

All blood samples were tested for HIV (anti-HIV 1/2), HBV (HBsAg, total anti-HBc, and anti-HBs), lifetime syphilis infection (antibody to T. pallidum), and HCV (anti-HCV) serological markers using the electrochemiluminescence (ECL) analyzed in the Cobas e601 Analyser (Roche Diagnostics, Mannheim, Germany). ECL-reactive samples for anti-*T*.*pallidum* were serially diluted to quantify Venereal Disease Research Laboratory (VDRL) titers and to determine whether the disease is active. As recommended by the Brazilian Ministry of Health, participants were considered to have active syphilis when they presented VDRL titers equal or superior to 1/8 plus treponemal test positive [18]. All positive and indeterminate specimens for antibodies against HIV-1/2 by ECL were confirmed by Western blot assay (Novopath HIV-I, Immunoblot, BioRad).

All positive or indeterminate samples for anti-HCV were submitted to the detection of HCV RNA by Real-Time HCV assay (qPCR) (Abbott RealTime HCV) and submitted to a direct nucleotide sequencing reaction in both directions using a Big Dye Terminator kit (version 3.1, Applied Biosystems, Foster City, CA, USA).

Data processing and analysis were performed in the statistical software Stata SE, version 13 (StataCorp LP, College Station, USA). We performed the chi-square test (χ2) to determine differences between subgroups with 95% confidence intervals (CIs). A 2-sided P-value < 0.05 was considered statistically significant.

## Results

The sociodemographic and clinical characteristics according to serological markers of exposure to HCV, HBV, HIV and syphilis infections among 279 participants are shown in Table 1 and supporting information–minimal data (DOI 10.6084/m9.figshare.8204525). The median age was 29 years. Of the total participants, 53.8% were multiracial, 59.5% had a non-steady partner and 63.8% were from MS State. The majority of participants had pulmonary TB manifestation (97.8%).

**Table 1:** Sociodemographic and clinical characteristics according to anti-HCV, total anti-HBc, anti-HIV, and anti-*T*.*pallidum* seropositivity among prisoners with bacteriologically confirmed tuberculosis in Central Brazil (n = 279).

	Anti-HCV positive		Total anti-HBc positive		Anti-HIV positive		Anti-*T*.*pallidum* positive	
	N	%	N	%	N	%	N	%
**Age (years)**								
<25 years (n = 211)	2	0.95	13	6.16	11	5.21	13	6.16
≥ 25 years (n = 68)	11	16.18	13	19.12	8	11.8	15	22.06
**Marital status**								
With a steady partner (n = 113)	3	2.65	7	6.19	4	3.54	7	6.19
Without a steady partner (n = 166)	10	6.02	19	11.45	15	9.04	21	12.65
**Race/ethnicity**								
White (n = 73)*	3	4.11	4	5.48	5	6.85	8	10.96
Multiracial (n = 150)	10	6.67	14	9.33	9	6.00	13	8.67
Black (n = 51)	0	0.00	8	15.69	5	9.80	7	13.73
Asian (n = 4)	0	0.00	0	0.00	0	0.00	0	0.00
**Education level (years)**								
≤4 (n = 68)	5	7.35	9	13.24	5	7.35	14	20.59
≥5 (n = 211)	8	3.79	17	8.06	14	6.64	14	6.64
**Naturality**								
MS (n = 178)	10	5.62	17	9.55	10	5.62	17	9.55
Others (n = 101)	3	2.97	9	8.91	9	8.91	11	10.89
**Type of tuberculosis disease**								
Pulmonary TB (n = 271)	13	4.80	23	8.49	16	5.90	25	9.23
Extra-pulmonary TB (n = 6)	0	0.00	3	50.0	3	50.0	2	33.33
**Penal institutions**								
EPJFC (n = 204)	9	4.41	19	9.31	8	3.92	18	8.82
IPCG (n = 75)	4	5.33	7	9.33	11	14.67	10	13.33

*Descendants of Europeans.

EPJC–Estabelecimento Penal Jair Ferreira de Carvalho; IPCG–Instituto Penal de Campo Grande; TB–Tuberculosis; MS–Mato Grosso do Sul State.

The overall prevalence of HCV exposure was 4.7% (13/279, 95% CI 2.2–7.1). Among the 279 participants, 19 (6.8%; 95% CI 4.4–10.4) were HIV co-infected, 1.4% (4/279, 95% CI 0.5–3.8) had chronic hepatitis B infection (HBsAg positive) and 9.3% (26/279, 95% CI 6.4–13.4) had serological marker of exposure to hepatitis B virus infection (total anti-HBc positive). The prevalence of lifetime syphilis infection (anti-*T*.*pallidum* positive with any VDRL titres) was 10% (28/279, 95% CI 7.0–14.2) and active syphilis (VDRL ≥ 1/8 titre) was 5% (14/279, 95% CI 2.9–8.3).

**Fig 1:**
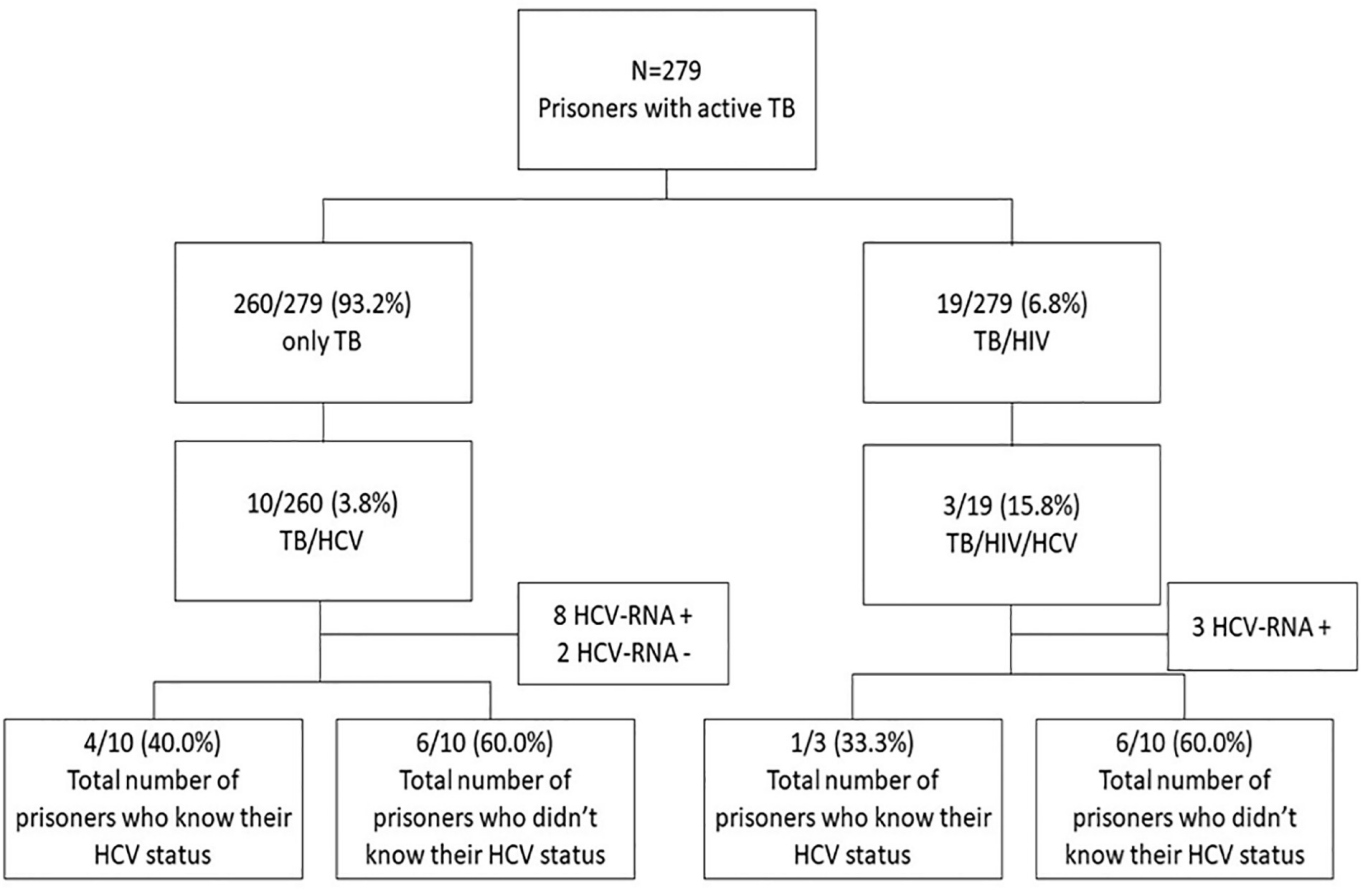
Enrollment of bacteriologically confirmed TB cases in the study.

Co-infection TB/HCV prevalence among participants with HIV (15.8%) was higher than that observed in HIV-non-infected inmates (3.8%; p<0.05) (Fig 1). Among all anti-HCV positive prisoners with bacteriologically confirmed TB, 23.1% (3/13) also presented positivity for anti-HIV-1, 23.1% (3/13) for anti-*Treponema pallidum*, and 30.8% (4/13) had serological evidence of prior HBV exposure (total anti-HBc positive).

Those with a serological marker of HCV, HBV and syphilis exposure were significantly more likely to be aged > 35 years (p<0.001; p = 0.002; p<0.001, respectively).

All anti-HIV-positive participants had access to HIV treatment, including antiretroviral therapy, care, and support. It was equivalent to that available to people living with HIV (PLWA) in the community and was in line with national guidelines. All bacteriologically confirmed TB prisoners with chronic hepatitis B infection had also access to hepatitis B treatment and tuberculosis programs, including treatment protocols [19, 20, 21]. During the study period, anti-HCV-positive participants did not receive the interferon-based HCV treatment often because all of them was not eligible for this treatment and also because of the difficulties in management and follow-up of this protocol [22]. Although benzathine penicillin is the recommended first-line treatment for syphilis in Brazil, participants with active syphilis were treated with an alternative antibiotic drug for the treatment of syphilis.

The presence of HCV RNA was detected in 11/13 (84.61%) of anti-HCV positive samples. Ten (90.9%) HCV RNA-positive samples were genotyped as genotype 1 and one (9.1%) as genotype 3.

## Discussion

The high-risk environment in prisons provides optimal conditions for acquiring and transmitting infectious diseases such as HBV, HCV and HIV exposure including TB [9]. The prevention and diagnosis of these infections among bacteriologically confirmed TB prisoners require immediate attention. Therefore, infectious diseases screening may provide benefits on treatment and subsequent monitoring of these co-infections.

Tuberculosis is still the most common opportunistic infection worldwide [4], and it has been endemic especially inside Brazilians prisons [23]. Anti-HCV prevalence rates are generally higher among patients with TB than in the general population [24, 25, 26]. In 2014, it was estimated that there were around 10.2 million prisoners worldwide, 2.8% had active TB, 15.1% had HCV infection, 3.8% had HIV and 4.8% had chronic HBV [3]. The prevalence of HCV exposure found in our study (4.7%) is consistent with previous studies conducted among prisoners in Mato Grosso do Sul state which reported 4.8% and 2.4% of HCV exposure in 2011 and 2017, respectively [27, 28]. These similar prevalence rates found in different periods of time may suggest that TB remains a persistent public health problem in a prison environment regardless of the clinical TB status of the prisoners.

The HCV prevalence found in this study (4.7%; 95% CI 2.2–7.1) was higher than that observed among the general population (1.38%) and higher than that previously reported among blood donors from the same region (0.17%) [29, 30]. This prevalence was lower than that found among imprisoned people from different countries, ranging from 8% to 95% [3]. Most of these rates are normally related to injection drug use [31, 32]. These variations may reflect regional differences in the prevalence of HCV infection and also could be explained by the low number of people who inject drugs (PWID) among Brazilian prisoners [23, 28]. In fact, PWID are more likely to be infected with HCV than non-PWID [25, 32].

Diagnosis of HIV seropositivity during the screening of tuberculosis is common [33]. In addition, among the HIV infected person, HCV co-infection is more prevalent due to overlapping transmission routes (sexual, parenteral and vertical) [34]. The prevalence of HCV infection was 4 times higher in TB/HIV-co-infected patients than in patients infected with TB only (15.8% vs. 3.8%; p<0.05). In fact, in our cohort of participants with bacteriologically confirmed TB, those with the serological marker of HCV exposure were significantly more likely to be anti-HIV positive, suggesting that HIV seropositivity may identify patients with unknown HCV infection.

Among bacteriologically confirmed TB prisoners (n = 11) chronically infected with HCV (HCV RNA positive), three were also coinfected with HIV. This clinical status may increase the risk of hepatotoxicity induced by the anti-TB drug during treatment [35]. The monitoring of treatment during the first 2 months should identify the Drug-induced liver injury, possibly shortening anti-TB treatment discontinuation and reducing mortality [36]. In addition, factors such as high mobility in and out of the prison environment, adverse effects of the anti-TB drug and use of illicit drug inside prisons may lead to TB treatment dropout.

Participants with evidence of HCV, HBV and syphilis exposure were significantly more likely to be aged > 35 years. Age is considered to be a cumulative risk factor for syphilis, HCV and HBV infections through blood, blood products, and sexual routes [28, 24, 37, 38].

Being transferred from establishments exposes the individual to a greater number of potentially infected persons, especially promoting high-risk sexual partnerships with several individuals, sharing of injecting equipment (needles and syringes) and reuse sharp and unsterile objects for tattooing and body piercing [39]. Once some of these behaviors were observed among participants with evidence of HCV, HBV and HIV exposure harm reduction efforts like condom use or needle exchange needs to be made available to the incarcerated population [40]. It is important to note that HCV RNA was detected in 84.61% of anti-HCV positive samples. Different studies have been reported HCV replication in anti-HCV positive participants (the presence of HCV RNA in serum) with a rate ranging from 45% to 90% [13, 17, 15, 16, 27, 41, 42]. This finding reinforces the importance of access to effective prevention and treatment strategies to reduce the transmission of this infection inside prisons.

Analysis of the data showed that genotype 1 is the predominant genotype circulating in prisoners with TB/HCV co-infection. This finding seems to be similar to the genotype pattern reported in the general population [30] and in the prisoners from the same region [27].

Co-infections have major clinical implications, since one infection may interfere with successful treatment of another or even potentiate a secondary effect of the medication. Therefore, according to Brazilian clinical protocols in TB co-infections with HCV, HIV and/or syphilis, the priority is always to treat HIV first to improve the patient’s immune response and the proportion of favorable therapeutic outcomes. Treatment of viral hepatitis is performed after treatment of TB and undetectable HIV viral load [19, 20, 21].

Jails are an ideal setting for HCV, HBV, syphilis and HIV infections screening and treatment not only because of the high frequency rates of specific risk behaviors which prisoners are exposed but also the potential risk of transmission inside prisons [28]. Therefore, it would be important to establish the routine of serological diagnosis of HCV infection, as well as other STI at the time of the arrival of new inmates and the continuous performance of tests during sentence completion [43]. It is important to note that the majority of inmates that were diagnosed with HCV infection in this study (53.8%) were unaware of their virological condition and also did not receive anti-HCV treatment, highlighting the need of implement strategies to improve the diagnostic and therapeutic approach to HCV in prisoners.

Although prison is an ideal setting for screening and treatment these infections, participants infected with HCV did not initiate treatment. The new guideline that including most HCV-infected patients for treatment with the new DAAs was published in 2019, prior to that, only more severe cases of liver damage, relapses, and HIV coinfection were treated. In addition, time and escort prisoners to obtain the results of genotyping, elastomeric and/or hepatic biopsy to classify the HCV-infected patient in inclusion criteria in the last guideline was a decisive factor in the treatment attempt [44]. The use of HCV DAAs therapy for chronic HCV infection that commonly requires no more than 12 weeks of therapy and causes few adverse effects is now logistically feasible within the prison setting and would aid the HCV elimination effort.

From 2014 to 2017, Brazil experienced a global problem that was the shortage benzathine penicillin, the first-choice drug for the treatment of active syphilis. Benzathine penicillin supply was insufficient in 17 of Brazil’s 27 states, including MS. Therefore, participants with active syphilis were treated with ceftriaxone, an alternative antibiotic drug for the treatment of syphilis that is longer, more expensive and less effective than benzathine penicillin [45, 46].

This study is subject to some limitations. Some potential risk factors associated with HCV, HBV, HIV and syphilis infections may have been under-reported due to the fear of punishment, which may have generated bias in data collection. Moreover, all participants were males, which limit the generalizability of the results to the female inmate population. Finally, we had insufficient numbers of events to examine risk factors for HBV, HCV, HIV and syphilis infections separately [47].

The present study confirmed that prisons represent a crucial setting for sexually transmitted infections, hepatitis C and tuberculosis control. Hence, it is necessary an integrated plan for reducing the spread of these infections in prisons by performing screening, intervention, and prevention and also improve the access to treatment, once an omission of those practices may represent a missed opportunity to control these infections inside and outside of prisons.

## Supporting information

S1 File Questionnaire. (DOCX)

S2 File Translated questionnaire. (DOCX)
