# Supplementary material for: Screening for HBV, HCV, HIV and syphilis infections among bacteriologically confirmed tuberculosis prisoners: An urgent action required
Source: PLoS One. 2019 Aug 22;14(8):e0221265. doi: 10.1371/journal.pone.0221265 (PMC6705821; doi:10.1371/journal.pone.0221265)
Supplement: S1 File — (DOCX) [file pone.0221265.s001.docx]

| **UNIVERSIDADE FEDERAL DE MATO GROSSO DO SUL**  **Fatores de Risco para HCV, HBV, HIV em coinfectados com Tuberculose na população carcerária de Campo Grande - Mato Grosso do Sul** |  | | |
| --- | --- | --- | --- |
| INFORMAÇÕES GERAIS | | | |
| 1. Número do questionário __ __ __ __  2. Responsável pela coleta de dados: __________________________  3. Data da coleta de dados: ____/____/_____  4. Digitador: ____________________  5. Data da digitação: ____/____/____  6. Cidade: ________________  7. Presídio: ____________________________  8. Pavilhão: __________________________  9. Identificação da cela: __________________  10. Identificação do participante (Iniciais do Nome): _______________  11. Sexo: __  12. Data de Nascimento: *___/___/___*  13. Cidade/Estados de origem:________  14. Qual sua cor ou raça? (1) Branca (2) Negra (3) Amarela (4) Parda  15. Estado civil: (1) Casado ou tem companheiro(a) (2) Viúvo(a) (3) Separado(a)/Divorciado(a) (4) Solteiro(a)  16. Qual foi a última série escolar que você cursou e foi aprovado? _____________  17. Trabalhava antes de ser preso? Tipo de trabalho?____________ | | *1.______*  *2.________*  *3.____/____/____*  *4.________*  *5.____/____/____*  *6._____________*  *7._____________*  *8.______*  *9.______*  *10._____________*  *11._____*  *12.____/____/____*  *13._____________*  *14.______*  *15.______*  *16.______*  *17.______* | |
| **DROGAS** | | | |
| 18. Qual seu peso? __ __ __  19. Qual sua altura? __ __ __  20. Você toma alguma medicação? __ **(1) Sim (2) Não. Se não, pular para a questão 22**  21. Se sim, especifique qual medicação faz uso? **_______________**  **Histórico de drogas e álcool**  22. Você fuma? __ **(1) Sim (2) Não**. **Se não, pular para a questão 25.**  23. Sem sim, quantos cigarros você fuma por dia? __ __  24. Se sim com que idade começou?_____  25.Você já fumou? (1) Sim (2) Não  **Você já usou alguma das seguintes drogas:**   \|  \| Você usou no último ano?  **(1) Sim**  **(2) Não** \| Quantas vezes você a usou?  **(1) Menos de uma vez na semana**  **(2) 1-2 vezes na semana**  **(3) + de 3 vezes na semana**  **(4) Todos os dias** \| Durante:  **(1) Dia**  **(2) Noite**  **(3) Os dois** \| Em:  **(1) Dias de semana**  **(2) Finais de semana**  **(3) Os dois** \| Você usou na prisão?  **(1) Sim**  **(2) Não** \| \| --- \| --- \| --- \| --- \| --- \| --- \| \| Álcool \| 26. \| 35. \| 44. \| 53. \| 62. \| \| Maconha \| 27. \| 36. \| 45. \| 54. \| 63. \| \| Cocaína \| 28. \| 37. \| 46. \| 55. \| 64. \| \| Crack (pedra) \| 29. \| 38. \| 47. \| 56. \| 65. \| \| Fumou heroína \| 30. \| 39. \| 48. \| 57. \| 66. \| \| Cheirou cola/ outros solventes \| 31. \| 40. \| 49. \| 58. \| 67. \| \| Pasta base \| 32. \| 41. \| 50. \| 59. \| 68. \| \| Haxixe \| 33. \| 42. \| 51. \| 60. \| 69. \| \| Injetou alguma droga? Quais: \| 34. \| 43. \| 52. \| 61. \| 70. \| | *18._____*  *19._____*  *20._____*  *21._____*  *22._____*  *23._____*  *24._____*  *25._____*  *26.___ 27.___ 28.___*  *29.___ 30.___ 31.___*  *32.___ 33.___ 34.___*  *35.___ 36.___ 37.___*  *38.___ 39.___ 40.___*  *41.___ 42.___ 43.___*  *44.___ 45.___ 46.___*  *47.___ 48.___ 49.___*  *50.___ 51.___ 52.___*  *53.___ 54.___ 55.___*  *56.___ 57.___ 58.___*  *59.___ 60.___ 61.___*  *62.___ 63.___ 64.___*  *65.___ 66.___ 67.___*  *68.___ 69.___ 70.___* | | |
| TUBERCULOSE | | | |
| **Histórico de sinais e sintomas relacionados a tuberculose**  71. Onde estava quando diagnosticado com tuberculose? ____________  72. Quando foi realizado o último tratamento?__ __ meses.  73. Esquema utilizado (o último):________________________________________  74. Tempo que usou a medicação (o último): __ __  75. Tipo de alta (a última): **__ (1) Cura (2) Abandono (3) em tratamento (4) Não sabe**  76. Você conheceu alguém com TB? __ **(1) Sim (2) Não (3) Não sabe. Se não, vá para a questão 78**  77. Você tem contato com essa pessoa? __ **(1) Menos de uma vez na semana (2) 1-2 vezes na semana**  **(3) + de 3 vezes na semana (4) Todos os dias**  78. Há pessoas na sua cela com tosse, febre ou emagreceu? __ **(1) Sim (2) Não**  79. Você tem tosse? **(1) Sim (2) Não. Se não vá para a questão 81.**  80. Por quantas semanas? __ __  81. Você tem expectoração? __ **(1) Sim (2) Não. Se não vá para a questão 84.**  82. Sua expectoração tem sangue? __ **(1) Sim (2) Não**  83. Por quantas semanas? __ __  84. Você tem febre? __ **(1) Sim (2) Não**  85. Você sente falta de apetite? __ **(1) Sim (2) Não**  86. Você emagreceu ou está emagrecendo? __ **(1) Sim (2) Não.**  87. Você tem sudorese noturna? __ **(1) Sim (2) Não. Se não vá para a questão 89**  88. Por quantas semanas ou dias? ___  89. Você sente dor torácica? __ **(1) Sim (2) Não**  90 Você sente dificuldade para respirar? __ **(1) Sim (2) Não**  91. Faz quanto tempo que você esta preso? _______  92. Já esteve preso antes? (1) Sim (2) Não. Por quanto tempo?______  93. No total por quanto tempo já esteve preso?______  94**.**Quanto tempo ficou em liberdade?_________  95 Você foi transferido quantas vezes de presídio? ______  96 Em quantas alas diferentes você passou neste presídio?  **___**  97 Em quantas celas você passou neste presídio?______  98. Qual o tamanho da cela? ___________  99. Quantas pessoas na sua cela?________  100.Você tem a marca da vacina BCG no braço direito? Posso ver? (1) Sim (2) Não | | | *71.____________*  *72.____________*  *73.____________*  *74.____________*  *75._____*  *76._____*  *77._____*  *78._____*  *79._____*  *80._____*  *81._____*  *82._____*  *83._____*  *84._____*  *85._____*  *86._____*  *87._____*  *88._____*  *89._____*  *90._____*  *91._____*  *92._____*  *93._____*  *94._____*  *95._____*  *96._____*  *97._____*  *98._____*  *99._____*  *100.____* |
| **DOENÇAS SEXUALMENTE TRANSMISSÍVEIS** | | | |
| 101. Você tem ou teve alguma doença sexualmente transmissível? __ **(1) Sim (2) Não (3) Não sabe**    102. Qual doença?__________________ **Se não vá para a questão 107.**  103. Qual o nome da medicação para o tratamento utilizado?__________________  104. Quantos tratamentos foram realizados? ___  105. Onde foi realizado o tratamento? ____________  106. Há quanto tempo foi realizado o último tratamento?____ meses.  107. Você tem HIV, HBV, HCV e/ou Sífilis? __ **(1) Sim (2) Não. Qual? ______**  108.Conhece as formas de transmissão da hepatite B e C? **(1) Sim (2) Não**  109. Você fez alguma transfusão sanguínea? __ **(1) Sim (2) Não. Se não, pular para a questão 111**  110. Se sim, em que ano?_________  111. Você tem tatuagem? (1) Sim (2) Não. Se sim, quantas? ______ **Se não, pular para a questão 113**  112. Tipo da tatuagem: **__ (1) caseira (2) profissional**  113. Você tem piercing? (1) Sim (2) Não. Se sim, quantos? _____  114. Trabalha ou já trabalhou como profissional do sexo? (1) Sim (2) Não  115. Você tem ou teve corrimento uretral? __ **(1) Sim (2) Não**    116. Você tem ou teve verruga no pênis ou vagina? __ **(1) Sim (2) Não**  117. Você tem alguma mancha na região palmar ou plantar?___**(1)Sim (2)Não**  118. Você tem ferida no pênis ou vagina? __ **(1) Sim (2) Não**  119. Você teve relação sexual com parceiro usuário de droga ilícita não-injetável? __  **(1) Sim (2) Não**  120. Você teve relação sexual com usuário de droga injetável?  **(1) Sim (2) Não**  121. Você teve relação sexual com parceiro com HIV? __  **(1) Sim (2) Não**  123. Tem parceiro sexual fixo? __  **(1) Sim (2) Não**  124. Se sim, há quantos tempo? __  125. Quanto tempo faz que teve a última relação sexual? ____meses  126. Qual a quantidade de parceiros no último ano? **__ __**  127. Qual sua orientação sexual? __ **(1) homossexual** **(2) heterossexual (3) bi**  128. Se for heterossexual, você já teve alguma relação homossexual? __ **(1) Sim (2) Não**  129. Você usa camisinha nas relações sexuais? **__ (1) Sempre (2) Ás vezes (3) Nunca**  130. Práticas sexuais frequentes? (1) Oral (2) Vaginal (3) Anal  131. Você já compartilhou seringas/agulhas? __  **(1) Sim (2) Não**  132. Você compartilhou objetos para realizar tatuagem, alicate, aparelho de barbear, para uso de droga inalatória? __  **(1) Sim (2) Não**  133. Realizou alguma cirurgia? __  **(1) Sim (2) Não. Se não, pular para a questão 135**  134. Se sim, em que ano?_______  135. Já tomou vacina da hepatite B? __  **(1) Sim (2) Não. Se não, pular para a questão 137.**  136. Se sim, quantas doses? __  137. Se gestante, qual semana de gestação?________**( ) Não se aplica**  138. Realizou o Pré-Natal? **(1) Sim (2) Não ( ) Não se aplica** | | | *101.____*  *102.____*  *103.____*  *104.____*  *105.____*  *106.____*  *107.____*  *108.____*  *109.____*  *110.____*  *111.____*  *112.____*  *113.____*  *114.____*  *115.____*  *116.____*  *117.____*  *118.____*  *119.____*  *120.____*  *121.____*  *122.____*  *123.____*  *124.____*  *125.____*  *126.____*  *127.____*  *128.____*  *129.____*  *130.____*  *131.____*  *132.____*  *133.____*  *134.____*  *135.____*  *136.____*  *137.____*  *138.____* |
| **EXAMES REALIZADOS** | | | |
| **Prova tuberculínica**  139. Realizada em: **__ (1) MSE (2) MSD**  140. Data: __/__/____ Horário: ___h ___min.  **Avaliação:**  141. Data da avaliação: ___/__/____ Horário: ___h ____min.  142. Resultado: _____ mm  **Escarro**  **1ª amostra**  143. Colhido: __ **(1) Sim (2) Não**  144. Data: __/__/____ Horário: ___h ____min.  145. Colhido em jejum: **__ (1) Sim (2) Não**  146. Resultado: ________________________________  **2ª amostra**  147. Colhido: __ **(1) Sim (2) Não**  148. Data: __/__/____ Horário: ___h ____min.  149. Colhido em jejum: **__ (1) Sim (2) Não**  150. Resultado: ________________________________  **Cultura**  151. Resultado: _______________________________  **Bioquímica**  **152.** AST:_______  **153.** ALT:_______  **Sorologias**  154. Data da coleta de sangue: **__/__/____**  155. HBsAg: __ **(1)Reagente (2) Não-reagente**  156. Anti-HBs: __ **(1)Reagente (2) Não-reagente**  157. Anti-HBc total: __ **(1)Reagente (2) Não-reagente**  158. Anti-HCV: __ **(1)Reagente (2) Não-reagente**  159. Anti-HIV 1/ 2: __ **(1)Reagente (2) Não-reagente**  160. Anti-Tpallidum____ **(1) Reagente (2) Não-reagente**  161. VDRL____ **(1)Reagente (2)Não Reagente**  162. Título:_____ | | | *139. __*  *140. __/__/____ _________*  *141. __/__/____ _________*  *142. __*  *143. __*  *144. __/__/____ _________*  *145. __*  *146. _______________*  *147. __*  *148. __/__/_____ ­­________*  *149. __*  *150. _______________*  *151. _______________*  *152.________*  *153.________*  *154. __/__/____*  *155. __*  *156. __*  *157. __*  *158. __*  *159. __*  *160. __*  *161. __*  *162.__* |
