# Supplementary material for: Screening for HBV, HCV, HIV and syphilis infections among bacteriologically confirmed tuberculosis prisoners: An urgent action required
Source: PLoS One. 2019 Aug 22;14(8):e0221265. doi: 10.1371/journal.pone.0221265 (PMC6705821; doi:10.1371/journal.pone.0221265)
Supplement: S2 File — (DOCX) [file pone.0221265.s002.docx]

| **FEDERAL UNIVERSITY OF MATO GROSSO DO SUL**  **Risk Factors for HCV, HBV, and HIV infection in prisoners coinfected with tuberculosis in prison population from Campo Grande - Mato Grosso do Sul** |  | | |
| --- | --- | --- | --- |
| GENERAL DATA | | | |
| 1. Questionnaire number __ __ __ __  2. Responsible for data collection: __________________________  3. Date of data collection: ____/____/_____  4. Digitizer: ____________________  5. Date of entry: ____/____/____  6. City: ________________  7. Prison: ____________________________  8. Pavilion: __________________________  9. Identification of the cell: __________________  10. Identification of the participant (Name Initials): _______________  11. Sex: __  12. Date of birth: *___/___/___*  13. City/State of origin:________  14. What is your color or race? (1) White (2) Black (3 ) Yellow (4) Brown  15. Marital Status: (1) Married or have steady partner (2) Widowed (3) Divorced (4) Single  16. What was the last grade you took and was approved? _____________  17. Had you worked before you get arrested? What kind of work? ____________ | | *1.______*  *2.________*  *3.____/____/____*  *4.________*  *5.____/____/____*  *6._____________*  *7._____________*  *8.______*  *9.______*  *10._____________*  *11._____*  *12.____/____/____*  *13._____________*  *14.______*  *15.______*  *16.______*  *17.______* | |
| **DRUGS** | | | |
| 18. What's your weight? __ __ __  19. What's your height? __ __ __  20. Do you take any medication? __ (1) Yes (2) No. If not, skip to question 22  21. If so, please specify which medication do you use? **______________________**  **History of drugs and alcohol**  22. Do you smoke? __ (1) Yes (2) No. If not, skip to the question 25.  23. If yes, how many cigarettes do you smoke per day?__ __  24. If so, at what age did you start smoking?_____  25. Have you ever smoked? (1) Yes (2) No  **Have you ever used any** **of the following drugs:**   \|  \| Did you use it last year?  ( 1 ) Yes  ( 2 ) No \| How many times have you used it?  **(** **1** **)** **Less than once a week**  **(2) 1-2 times a week**  **(** **3** **)** **+ 3 times a week**  **(** **4** **)** **Every day** \| Period of the day you used the drug:  **(1) Day**  **(2) Night**  **(3) Both** \| On:  **(1) Weekdays**  **(2) Weekend**  **(3) Both** \| Did you use it in prison?  (1) Yes  (2) No \| \| --- \| --- \| --- \| --- \| --- \| --- \| \| Alcohol \| 26. \| 35. \| 44. \| 53. \| 62. \| \| Marijuana \| 27. \| 36. \| 45. \| 54. \| 63. \| \| Cocaine \| 28. \| 37. \| 46. \| 55. \| 64. \| \| Crack (stone) \| 29. \| 38. \| 47. \| 56. \| 65. \| \| Smoked heroin \| 30. \| 39. \| 48. \| 57. \| 66. \| \| Chewed glue/ other solvents \| 31. \| 40. \| 49. \| 58. \| 67. \| \| Cocaine paste \| 32. \| 41. \| 50. \| 59. \| 68. \| \| Hashish \| 33. \| 42. \| 51. \| 60. \| 69. \| \| Did you inject any drugs? Which: \| 34. \| 43. \| 52. \| 61. \| 70. \| | *18._____*  *19._____*  *20._____*  *21._____*  *22._____*  *23._____*  *24._____*  *25._____*  *26.___ 27.___ 28.___*  *29.___ 30.___ 31.___*  *32.___ 33.___ 34.___*  *35.___ 36.___ 37.___*  *38.___ 39.___ 40.___*  *41.___ 42.___ 43.___*  *44.___ 45.___ 46.___*  *47.___ 48.___ 49.___*  *50.___ 51.___ 52.___*  *53.___ 54.___ 55.___*  *56.___ 57.___ 58.___*  *59.___ 60.___ 61.___*  *62.___ 63.___ 64.___*  *65.___ 66.___ 67.___*  *68.___ 69.___ 70.___* | | |
| TUBERCULOSIS | | | |
| **History of signs and symptoms related to tuberculosis**  71. Where were you when you were diagnosed with tuberculosis?____________  72. When was the last treatment completed? __ __ months.  73. Scheme used (the last one): _______________________________________  74. How long have you used the medicines (last treatment): __ __  75. Type of discharge (last ): __ ( 1 ) Cure ( 2 ) Abandonment (3) under treatment ( 4 ) Do not know  76. Do you know someone with TB? __   **(1) Yes (2) No (3) Do** **not know.** **If not, go to question** **78**  77. Do you have contact with this person? __   **(1) Less than** **once** **a** **week (2) 1-2 times a week**  **(3) + 3 times a week (4) Every day**  78. Are there people in your cell with a cough, fever, or weight loss? __ (1) Yes (2) No  79. Are you coughing?  **(1) Yes (2) No. If you do not go to question** **81.**  80. For how long (weeks) ? __ __  81. Do you have expectoration? __ **(1) Yes (2) No. If you do not go to the question** **84.**  82. Does your sputum have blood? __ (1) Yes (2) No  83. How long (weeks) ? __ __  84. Do you have fever? **__ (1) Yes (2) No**  85. Are you lacking in appetite?  **(1) Yes (2) No**  86. Have you lost weight or are you losing weight? __ **(1) Yes (2) No**  87. Do you have night sweats? __    **(1) Yes (2) No. If you do not go to the question** **89**  88. How long? ___  89. Do you have chest pain? __ **(1) Yes** **(2) No**  90 Do you have difficulty breathing? __ **(1) Yes (2) No**  91. How long have you been in jail? _______  92. Have you been in jail before? (1) Yes (2) No. For how long? ______  93. How long, in total, have you ever been in jail? ________(months)  94**.**  How long have you been released from prison?_________  95 How many times have you been transferred between prisons?_____  96 How many different pavilions did you get arrested in this prison??  **___**  97 In how many cells have you been in this prison?______  98. How big is the cell, in square meters? ___________  99. How many people are in your cell?________  100. Do you have the BCG vaccine brand on your right arm? Can I see? (1) Yes (2) No | | | *71.____________*  *72.____________*  *73.____________*  *74.____________*  *75._____*  *76._____*  *77._____*  *78._____*  *79._____*  *80._____*  *81._____*  *82._____*  *83._____*  *84._____*  *85._____*  *86._____*  *87._____*  *88._____*  *89._____*  *90._____*  *91._____*  *92._____*  *93._____*  *94._____*  *95._____*  *96._____*  *97._____*  *98._____*  *99._____*  *100.____* |
| **SEXUALLY TRANSMITTED DISEASES** | | | |
| 101. Do you have or have you had any sexually transmitted diseases? __ (1) Yes (2) No (3) Do not know    102. Which disease?__________________ **If you do not go to question** **107.**  103. What medicine did you take to treat it?__________________  104. How many treatments have been performed? ______  105. Where was the treatment performed ? _________________________  106. How long has the last treatment taken?____ months.  107. Do you have HIV , HBV, HCV and / or syphilis infection? __ **(1) Yes (2) No. Which? ______**  108. Do you know the pathways of transmission of hepatitis B and C?  **(1) Yes (2) No**  109. Have you received any blood transfusions? __ **(1) Yes (2) No.** **If not, skip to question** **111**  110. If yes, when was it?_________  111. Do you have any tattoo? (1) Yes (2) No. If yes, how many? __ ____ **If not, skip to question 113**  112. Type of tattoo: __ (1) homemade (2) professional  113. Do you have any body piercing? (1) Yes (2) No. If yes, how many? _____  114. Do you work or have you ever worked as a sex worker? (1) Yes (2) No  115. Have you had or had urethral discharge? __ **(1) Yes (2) No**    116. Do you have or have had a wart on the penis or vagina? __ **(1) Yes (2) No**  117. Do you have any spots on the palm or sole? ___ **(1) Yes (2) No**  118. Do you have a sore on the penis or vagina? __ **(1) Yes (2) No**  119. Have you ever had sexual intercourse with a non-injecting drug user? __ **(1) Yes (2) No**  120. Have you ever had sexual intercourse with an injecting drug user? __ **(1) Yes (2) No**  121. Have you ever had sex with an HIV-infected partner? __  **(1) Yes (2) No**  123. Do you have a steady sexual partner? __ **(1) Yes (2) No**  124. If so, for how long ? ____  125. How long have you had last sexual intercourse? ____months  126. How many sexual partners have you had in the last year?_____**__ __**  127. What is your sexual orientation? __ **(1) homosexual (2) heterosexual (3) bisexual**  128. If you are heterosexual, have you ever had a homosexual relationship? __ **(1) Yes (2) No**  129. How often do you use condoms during sex?**__**  **(1) Always (2) Sometimes (3) Never**  130. What are the most common sexual practices? **(1) Oral (2) Vaginal (3) Anal**  131. Have you ever shared syringes / needles? **__ (1) Yes (2) No**  132. Have you ever shared objects to perform tattooing, pliers, razors, for inhaled drug use? _**_ (1) Yes (2) No**  133. Have you ever had any surgery? __ **(1) Yes (2) No.** **If not, skip to question** **135**  134. If yes, when?_______  135. Have you ever had hepatitis B vaccine**? __ (1) Yes (2) No. If not, skip to question 137.**  136.  If yes, how many doses? __  137. Are you pregnant? If so, which week of gestation?________**( ) Not applicable**  138. Did you have prenatal care? **(1) Yes (2) No ( ) Not applicable** | | | *101.____*  *102.____*  *103.____*  *104.____*  *105.____*  *106.____*  *107.____*  *108.____*  *109.____*  *110.____*  *111.____*  *112.____*  *113.____*  *114.____*  *115.____*  *116.____*  *117.____*  *118.____*  *119.____*  *120.____*  *121.____*  *122.____*  *123.____*  *124.____*  *125.____*  *126.____*  *127.____*  *128.____*  *129.____*  *130.____*  *131.____*  *132.____*  *133.____*  *134.____*  *135.____*  *136.____*  *137.____*  *138.____* |
| **MECICAL EXAMS** | | | |
| **Tuberculin test**  139. Done in: **__ (1) MSE (2) MSD**  140. Date: __/__/____ Time: ___h ___min.  **Evaluation:**  141. Date of evaluation: ___ / __ / ____ Time: ___h ____min.   142. Result: _____ mm    **Sputum**  **1st sample**  143. Obtained: __ **(1) Yes (2) No**  144. Date: __/__/____ Time: ___h ____min.  145. Obtained from fasting patient : **__ (1) Yes (2) No**  146. Result: ________________________________  **2nd sample**  147. Obtained: __ **(1) Yes (2) No**  148. Date: __/__/____ Time: ___h ____min.  149. Obtained from fasting patient : **__ (1) Yes (2) No**  150. Result: ________________________________  **Culture**  151. Resultad: _______________________________  **Biochemical exams**  **152.** AST:_______  **153.** ALT:_______  **Serology**  154. Date of blood collection : **__/__/____**  155. HBsAg: __ **(1) Reagent (2) Non-reagent**  156. Anti-HBs: __ **(1) Reagent (2) Non-reagent**  157. Total Anti-HBc: __ **(1) Reagent (2) Non-reagent**  158. Anti-HCV: __ **(1) Reagent (2) Non-reagent**  159. Anti-HIV 1/ 2: __ **(1) Reagent (2) Non-reagent**  160. Anti-Tpallidum____ **(1) Reagent (2) Non-reagent**  161. VDRL____ **(1) Reagent (2) Non-reagent**  162. Title:_____ | | | *139. __*  *140. __/__/____ _________*  *141. __/__/____ _________*  *142. __*  *143. __*  *144. __/__/____ _________*  *145. __*  *146. _______________*  *147. __*  *148. __/__/_____ ­­________*  *149. __*  *150. _______________*  *151. _______________*  *152.________*  *153.________*  *154. __/__/____*  *155. __*  *156. __*  *157. __*  *158. __*  *159. __*  *160. __*  *161. __*  *162.__* |
